# Serotonergic Modulation of Sensory Neuron Activity and Behavior in *Apteronotus albifrons*

**DOI:** 10.3389/fnint.2020.00038

**Published:** 2020-07-07

**Authors:** Mariana M. Marquez, Maurice J. Chacron

**Affiliations:** Computational Systems Neuroscience Laboratory, Department of Physiology, McGill University, Montreal, QC, Canada

**Keywords:** neuromodulation, weakly electric fish, sensory processing, envelope, comparative

## Abstract

Organisms must constantly adapt to changes in their environment to survive. It is thought that neuromodulators such as serotonin enable sensory neurons to better process input encountered during different behavioral contexts. Here, we investigated how serotonergic innervation affects neural and behavioral responses to behaviorally relevant envelope stimuli in the weakly electric fish species *Apteronotus albifrons*. Under baseline conditions, we found that exogenous serotonin application within the electrosensory lateral line lobe increased sensory neuron excitability, thereby promoting burst firing. We found that serotonin enhanced the responses to envelope stimuli of pyramidal cells within the lateral segment of the electrosensory lateral line lobe (ELL) by increasing sensitivity, with the increase more pronounced for stimuli with higher temporal frequencies (i.e., >0.2 Hz). Such increases in neural sensitivity were due to increased burst firing. At the organismal level, bilateral serotonin application within the ELL lateral segment enhanced behavioral responses to sensory input through increases in sensitivity. Similar to what was observed for neural responses, increases in behavioral sensitivity were more pronounced for higher (i.e., >0.2 Hz) temporal frequencies. Surprisingly, a comparison between our results and previous ones obtained in the closely related species *A. leptorhynchus* revealed that, while serotonin application gave rise to similar effects on neural excitability and responses to sensory input, serotonin application also gave rise to marked differences in behavior. Specifically, behavioral responses in *A. leptorhynchus* were increased primarily for lower (i.e., ≤0.2 Hz) rather than for higher temporal frequencies. Thus, our results strongly suggest that there are marked differences in how sensory neural responses are processed downstream to give rise to behavior across both species. This is even though previous results have shown that the behavioral responses of both species to envelope stimuli were identical when serotonin is not applied.

## Introduction

Organisms must detect and perceive natural stimuli efficiently and adaptively to survive in ever-changing environments (Wark et al., 2007; Sharpee et al., 2014). Serotonergic fibers originating from the raphe nuclei are found across sensory brain areas in all vertebrate species (Parent, 1981; Hurley et al., 2004) and are thought, in part, to help mediate adaptive sensory neural responses to changes in input (Marder, 2012). Here, we used a comparative approach to better understand the function of the serotonergic system in sensory systems. Specifically, we investigated how serotonin application affects neural and behavioral responses to sensory input in the weakly electric fish species *Apteronotus albifrons* using the same paradigms used previously by our group (Marquez and Chacron, 2020b) in the closely related species *Apteronotus leptorhynchus*.

Gymnotiform weakly electric fish generate a quasi-sinusoidal electric field through their electric organ discharge (EOD) and can sense amplitude modulations of this field through an array of electroreceptor afferents embedded in their skin (Turner et al., 1999). Electroreceptor afferents make synaptic contact with pyramidal cells within the electrosensory lateral line lobe (ELL), which in turn project to higher brain centers mediating behavioral responses (Rose, 2004). Natural electrosensory stimuli comprise those caused by conspecifics. Specifically, when two fish are located close to one another (i.e., <1 m apart), interference between their EODs gives rise to sinusoidal amplitude modulation (AM) whose frequency is given by the difference between the EOD frequencies. Changes in the relative orientation and distance between both animals give rise to changes in the amplitude (i.e., the envelope) of the sinusoidal amplitude modulation (Yu et al., 2012; Fotowat et al., 2013; Metzen and Chacron, 2014; Huang et al., 2019). As such, the frequency components of the envelope are directly associated with the statistics of the relative movement between fish. The responses of ELL pyramidal cells to envelope stimuli have been well-characterized in both *A. leptorhynchus* (Huang and Chacron, 2016; Huang et al., 2016, 2018; Metzen et al., 2018; for review see Huang and Chacron, 2017; Metzen and Chacron, 2019) and *A. albifrons* (Martinez et al., 2016). Particularly, it has been shown that the tuning of ELL pyramidal cells to envelope stimuli is similar in both species. Notably, both *A. leptorhynchus* and *A. albifrons* display identical behavioral responses to envelope stimuli in that the animal’s EOD frequency follows the detailed timecourse of the envelope in an almost one-to-one fashion (Metzen and Chacron, 2014, 2015; Huang et al., 2016; Martinez et al., 2016; Thomas et al., 2018).

ELL pyramidal cells also receive large amounts of descending input (for review see Hofmann and Chacron, 2019) including serotonergic innervation (Johnston et al., 1990; Deemyad et al., 2011; Fotowat et al., 2016; for review see Márquez et al., 2013; Marquez and Chacron, 2020a). Previous studies carried out in *A. leptorhynchus* have shown that serotonin increases pyramidal neuron excitability by promoting burst firing through inhibition of potassium channels (Deemyad et al., 2011, 2013; Larson et al., 2014; Marquez and Chacron, 2020b). In the case of envelopes, it was shown in *A. leptorhynchus* that serotonin application increases neural sensitivity to these stimuli, with greater increases in sensitivity for higher temporal frequencies, due to an increase in burst firing during stimulation. At the organismal level, it was shown that serotonin application increases behavioral sensitivity to envelopes, but that there was a greater increase in sensitivity for lower temporal frequencies (Marquez and Chacron, 2020b). In contrast, how serotonin affects neural and behavioral responses in *A. albifrons* has not been extensively characterized. Although it was recently shown that serotonin application increases pyramidal neuron excitability through increased burst firing, which enhances their responses to moving objects (Marquez and Chacron, 2018), how serotonin application affects neural and behavioral responses to envelope stimuli has not been investigated in *A. albifrons*.

Here, we used both electrophysiological recordings and behavioral assays to investigate how serotonin application alters ELL pyramidal cell and behavioral responses to envelope stimuli in *A. albifrons*. Our results show that serotonin application increases ELL pyramidal neuron excitability through increased burst firing under baseline conditions (i.e., in the presence of the animals unmodulated EOD). Serotonin application increased ELL pyramidal cell responses to envelope stimuli, with greater increases seen for high temporal frequencies. At the organismal level, serotonin application increased behavioral sensitivity, such that envelope stimuli gave rise to greater modulations in EOD frequency. Unexpectedly, such increases in behavioral sensitivity were greatest for higher temporal frequencies. We discuss the implications of our results in the context of previous ones obtained for *A. leptorhynchus*.

## Materials and Methods

We note that the purpose of this study was to investigate how serotonin affected both ELL pyramidal cell and behavioral responses in *A. albifrons* and to compare these results with those previously obtained in *A. leptorhynchus*. As such, the methodology used in this article is the same as that used previously (Marquez and Chacron, 2020b), except that the experiments were performed in a different species. We used wording similar to that found within the methods section of Marquez and Chacron (2020b) to facilitate the comparison between both studies.

### Ethics Statement

All animal care and experimental procedures were reviewed and approved by McGill University’s animal care committee under protocol number 5285.

### Animals

We used a total of *N* = 26 *A. albifrons* specimens of either sex in this study. Animals were acquired from tropical fish suppliers and acclimated to laboratory conditions according to published guidelines (Hitschfeld et al., 2009).

### Surgery and Recordings

Surgical procedures have been described in detail previously (Deemyad et al., 2013; Marquez and Chacron, 2018). Briefly, tubocurarine chloride hydrate (0.1–0.5 mg) was injected to immobilize animals (*N* = 26). These were then transferred to an experimental tank and we used a constant flow of water over the gills (~10 ml/min) for respiration. A portion of the animal’s head was kept out of the water and anesthetized locally with lidocaine ointment (5%). Then, to expose the hindbrain for recording, a small craniotomy (~5 mm^2^) was made above the hindbrain for recordings. Extracellular recordings from pyramidal cells within the lateral segment (*n* = 16), based on recording depth and mediolateral positioning of the electrodes, were performed with micropipettes filled with Woods Metal alloy (LMA-117, Small Parts Inc) following standard methodology (Frank and Becker, 1964). The sample sizes are similar to those used in previous studies. Recordings were digitized at 10 kHz (CED Power 1401 and Spike 2 software, Cambridge Electronic Design) and stored on a computer for subsequent analysis. The population-averaged baseline (i.e., when the animal’s EOD was not modulated) firing rate was 11.38 ± 5.78 Hz and was similar to that reported in previous studies (Martinez et al., 2016; Marquez and Chacron, 2018).

Action potential times were defined as the times at which the signal crossed a suitably chosen threshold value. Baseline statistics (e.g., firing rate) were obtained from 100 s of baseline activity before stimulus presentation. A burst threshold corresponding to the trough of the bimodal interspike interval (ISI) distribution (typically 10 ms) was used to separate the full spike train into the burst train and the isolated spike train, as done previously (Oswald et al., 2004; Ellis et al., 2007; Khosravi-Hashemi et al., 2011; Khosravi-Hashemi and Chacron, 2012, 2014; Deemyad et al., 2013; Marquez and Chacron, 2018). Specifically, if an ISI was less than the threshold, then the two action potentials were deemed to be part of a burst; if the next ISI was also less than the threshold, then the third action potential was also deemed to be part of the same burst. This process continues until the ISI is greater than the threshold. The isolated spike train consists of spikes that were not part of bursts. Burst fraction was defined as the ratio of the number of spikes that belong to a burst to the total number of spikes.

To visualize neural responses to envelopes, we created a binary sequence *R(t)* with binwidth Δ*t* = 0.1 ms from the spike time sequence and set the content of each bin to equal the number of spikes which fell within that bin. Time-dependent firing rates were obtained by low-pass filtering the binary sequences using a Kaiser filter whose cutoff frequency was 0.1% higher than the envelope frequency (see below).

To quantify neural responses to envelopes, we used linear systems identification techniques. Specifically, the neural gain is defined as the ratio of the amplitude of the modulated firing rate response and the amplitude of the stimulus obtained from a dipole in the water. To determine the firing rate modulation, we computed the phase histogram and fitted a sinewave to it as done previously (Marquez and Chacron, 2020b). The response phase was calculated as the average phase at which the fitted sinewave reached its maximum value relative to the maximum value of the stimulus waveform.

### Stimulation

The neurogenic electric organ of *A. albifrons* is not affected by the injection of curare-like drugs. Stimuli consisted of amplitude modulations of the animal’s own EOD and were produced by first detecting the EOD zero crossings, then generating a sinusoidal waveform train with a frequency slightly greater (20–30 Hz) than the EOD frequency that is triggered by the EOD zero crossings. This train is thus synchronized to the fish’s EOD and will either increase or decrease the EOD amplitude based on polarity and intensity. This train is then multiplied (MT3 multiplier, Tucker Davis Technologies) with an amplitude modulated waveform (i.e., the stimulus). The resultant signal is then isolated from ground (A395 linear stimulus isolator, World Precision Instruments) and delivered to the experimental tank *via* two chloridized silver wire electrodes located ~15 cm on each side of the animal (Bastian et al., 2002). To elicit neural and behavioral responses to envelopes, we used band-pass filtered noise (5–15 Hz, 4th order Butterworth) whose amplitude (i.e., the envelope) was modulated sinusoidally at 0.05, 0.1, 0.2, 0.5, 0.75, and 1 Hz. This stimulus structure is the same as that used previously in *A. leptorhynchus* (Marquez and Chacron, 2020b). We used a low pass filtered Gaussian white noise stimulus and zero mean (8th order Butterworth filter, 120 Hz cutoff frequency) to distinguish between cell types as described below. We also used a 4 Hz sinusoidal amplitude modulation to elicit behavioral responses (see below). Stimulus intensity was adjusted to produce changes in EOD amplitude that were ~25% of the baseline level.

### Behavior

We first recorded the jamming avoidance response (JAR) in response to a 4 Hz sinusoidal AM stimulation as mentioned above. The JAR magnitude was defined as the maximum EOD frequency elicited during stimulation relative to the EOD frequency baseline value (i.e., before stimulation). JAR responses were averaged across five stimulus presentations of 50 s each and compared before and after serotonin application as done previously (Deemyad et al., 2013; Marquez and Chacron, 2020b). Behavioral responses to sinusoidal envelopes were quantified using linear systems identifications techniques (Metzen and Chacron, 2014). Thus, the gain was defined as the ratio of the EOD frequency peak-to-peak amplitude to that of the envelope stimulus, the phase is the amount of time relative to the envelope cycle that EOD frequency must be shifted by to be in phase with the sinusoidal envelope stimulus, and the offset is the difference between the mean EOD frequency during stimulation and the EOD frequency baseline value.

### Serotonin Application

Glutamate (1 mM; Sigma-Aldrich) and serotonin (1 mM; Sigma-Aldrich) were dissolved in saline (111 mM NaCl, 2 mM KCl, 2 mM CaCl_2_, 1 mM MgSO_4_, 1 mM NaHCO_3_ and 0.5 mM NaH_2_PO_4_; Sigma-Aldrich) for application. Drug application electrodes were made using either two-barrel (for electrophysiology) or single-barrel (for behavior) glass micropipettes as described previously (Huang et al., 2018, 2019; Marquez and Chacron, 2018, 2020b; Metzen et al., 2018). For single neuron recordings, two-barrel pipettes were used for independent application of serotonin or glutamate in the vicinity of the neuron being recorded. We relied on glutamate-elicited excitatory responses to verify that the pipette was correctly placed next to the neuron we were recording from, as done previously (Bastian, 1993; Marquez and Chacron, 2020b). For behavioral experiments, single-barrel pipettes were used for the bilateral application of serotonin in the lateral segment of the ELL. Drugs were delivered using a picospritzer at 15–25 p.s.i. during 100 ms, as done previously (Deemyad et al., 2013; Huang et al., 2018; Marquez and Chacron, 2018, 2020b). We note that previous studies have shown that the application of saline alone in this manner does not significantly alter either ELL pyramidal cell activity or behavior (Deemyad et al., 2013; Huang et al., 2016).

### Classification of Cell Types

Previous studies have shown that there are two types of ELL pyramidal cells, while ON-type cells respond with excitation to increases in EOD amplitude, OFF-type cells instead respond with excitation to decreases in EOD amplitude (Saunders and Bastian, 1984; Martinez et al., 2016). We used a low pass filtered Gaussian white noise stimulus and zero mean (8th order Butterworth filter, 120 Hz cutoff frequency) as an amplitude modulation to distinguish between ON- and OFF-type ELL pyramidal cells, as described previously (Martinez et al., 2016). Specifically, we computed the spike-triggered average (STA) by averaging stimulus segments during 1 s windows centered at the action potential times. Thus, the STA is given by $\frac{1}{N}\sum_{\left(i=1\right)}^{N}S(t-t_{i})$, where *S*(*t*) is the 0–120 Hz stimulus, *t*_i_ is the *i*th spike time, and *N* is the total number of action potentials. The average STA slope within a time window of 10 ms centered at 7 ms before the action potential time was used to distinguish between ON- and OFF-type cells. The 7 ms accounts for the transmission delay from the skin surface to pyramidal cells within the ELL (Chacron et al., 2003). Cells for which slope was positive were classified as ON-type and cells for which the slope was negative were classified as OFF-type (Martinez et al., 2016). Confirming previous results by Martinez et al. (2016), we found no differences between the responses of ON- and OFF-type cells to envelope stimuli before serotonin application. Moreover, we found no differences in the effects of serotonin on either the baseline activity of ON- and OFF-type ELL pyramidal cells (change in burst fraction: ON-type 0.17 ± 0.16, OFF-type 0.22 ± 0.23, Kruskal–Wallis test, chi-square = 0.33, *df* = 1, *P* = 0.56; change in firing rate: ON-type 5.25 ± 5.05 Hz, OFF-type 4.71 ± 1.28 Hz, Kruskal–Wallis test, chi-square = 0.89, *df* = 1, *P* = 0.34), or the change in sensitivity (ON-type: change in sensitivity 3.03 ± 3.57 (spk/s)/(mV/cm); OFF-type change in sensitivity 4.74 ± 10.66 (spk/s)/(mV/cm); Kruskal–Wallis test, chi-square = 0.71, *df* = 1, *P* = 0.40), best-fit power law exponent to neural tuning curve (ON-type: change in α exponent 0.34 ± 0.33; OFF-type change in α exponent 0.17 ± 0.35; Kruskal–Wallis test, chi-square = 2.48, *df* = 1, *P* = 0.12) and phase (ON-type: change in phase −9.50 ± 16.09 deg; OFF-type change in phase −2.40 ± 20.22 deg; Kruskal–Wallis test, chi-square = 0.80, *df* = 1, *P* = 0.37) during stimulation. For these reasons, data from ON- and OFF-type cells were pooled.

### Statistics

All values are reported as means ± SD throughout. Statistical significance was evaluated through either a parametric Student’s *t*-test or a non-parametric Wilcoxon’s signed-rank test for paired measurements at the *P* = 0.05 level. The choice of the test was based on: (1) whether the data followed a normal distribution (parametric test) or not (non-parametric test), as assessed by a Lilliefors test; and (2) whether the data had the same variance, as asses by an *F*-test. For multiple comparisons of phase and offset values, statistical significance was assessed through a Kruskal–Wallis test at the *P* = 0.05 level, since all the values followed a not normal distribution as assessed by a Lilliefors test. Correlations were calculated using Pearson’s correlation coefficient. For the whisker boxplots, the central mark indicates the median, and the bottom and top edges indicate the 25th and 75th percentiles, respectively. Whiskers extend to the values that are not considered outliers. All data points including outliers are plotted. To improve the readability of data points in some figures, we plotted compact box plots that include the median, the bottom, and top edges only.

## Results

**Figure 1:**
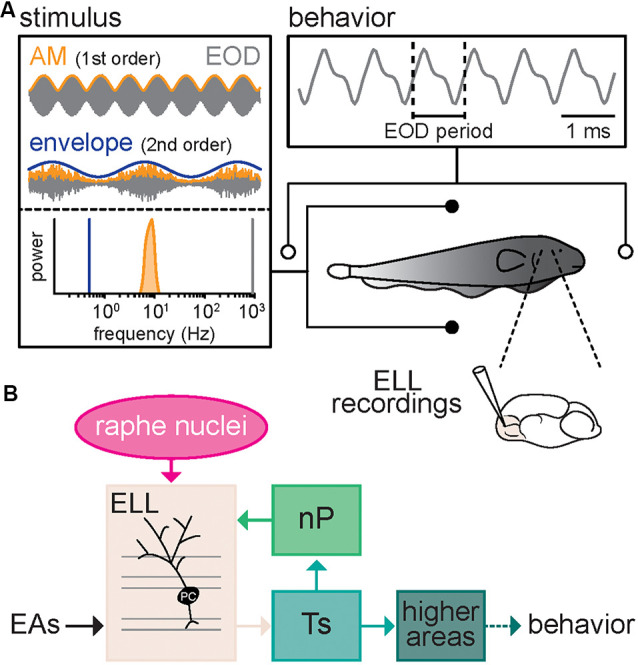
Schematic of the experimental setup. **(A)** Schematic showing the recording setup. A fish is placed in a tank while its electric organ discharge (EOD) is being recorded *via* two electrodes located near the animal’s head and tail. The neural responses consist of single-unit recordings from pyramidal neurons within the electrosensory lateral line lobe (ELL). The behavioral responses consist of changes in the frequency (i.e., the inverse of the period) of the quasi-sinusoidal EOD signal. The stimuli consist of amplitude modulations (AMs, orange) of the animal’s own EOD (gray) that were either a 4 Hz sinewave (top, orange, AM or 1st order) or 5–15 Hz noise (bottom, orange, AM or 1st order) whose amplitude (bottom, blue, envelope or 2nd order) varied sinusoidally. Also shown are the frequency contents of the envelope (blue), AM (orange), and full signal (gray). **(B)** Schematic showing successive brain areas involved in the processing of electrosensory stimuli. Electroreceptor afferents (EAs) respond to EOD AMs and project to pyramidal cells (PC) within the ELL. ELL pyramidal cells in turn project to the midbrain Torus semicircularis (Ts) and indirectly to higher brain areas, thereby mediating behavioral responses. ELL pyramidal cells also receive descending projections from higher brain centers *via* the nucleus praeeminentialis (nP), as well as serotonergic fibers from the raphe nuclei (magenta).

We investigated how serotonin altered both neural and behavioral responses to envelope stimuli. To do so, we recorded neural activity as well as behavioral responses, which consist of changes in the EOD frequency, from immobilized animals in a tank while presenting different stimuli (Figure 1A). Our recordings were from pyramidal cells within the lateral segment (LS) of ELL that receives neuromodulatory input from the raphe nuclei (Figure 1B).

### Serotonin Application Increases ELL Pyramidal Cell Excitability Through Increased Burst Firing

**Figure 2:**
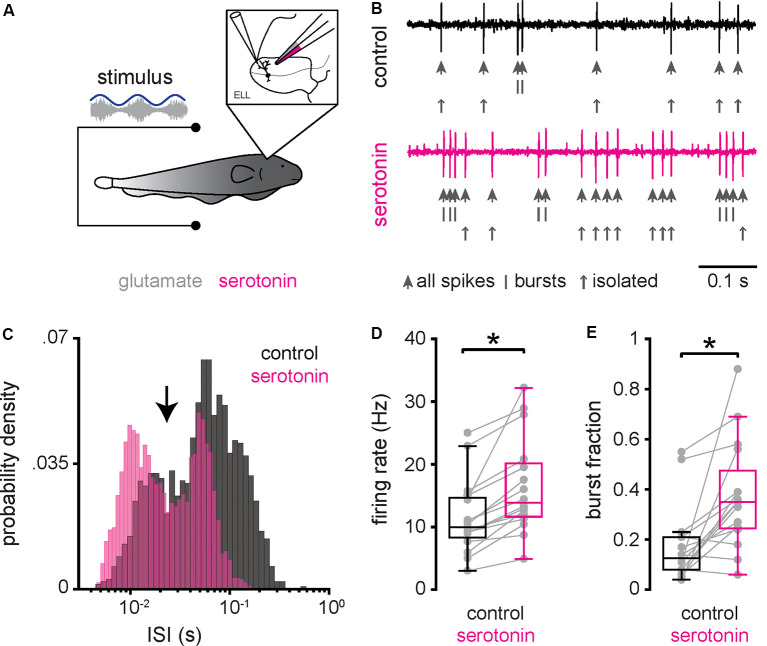
Serotonin application increases ELL pyramidal neuron excitability and promotes burst firing under baseline conditions. **(A)** Schematic showing a pyramidal cell that is being recorded from extracellularly while a double-barrel pipette containing both glutamate and serotonin is located in the vicinity. Glutamate is used to ascertain that the double-barrel pipette is close to the pyramidal cell being recorded from as judged from short-latency increases in firing rate following glutamate release *via* air pressure (Bastian, 1993; Marquez and Chacron, 2018; Marquez and Chacron, 2020b). Serotonin is then applied *via* air pressure (see “Materials and Methods” section). **(B)** Extracellular recording from a typical pyramidal cell before (top, black) and after (bottom, magenta) application of serotonin. In each case, the action potentials are indicated by large vertical arrows, while the spikes belonging to bursts and isolated spikes (i.e., spikes not belonging to bursts) are indicated by vertical bars and small vertical arrows, respectively. **(C)** Interspike interval (ISI) distribution from this same example pyramidal neuron before (black) and after (magenta) serotonin application. Note the bimodal distribution. An ISI threshold corresponding to the trough between both modes (vertical arrow) was used to separate the spike train into bursts and isolated spikes (see “Materials and Methods” section). **(D)** Firing-rate before (left, black) was significantly lower than that after (right, magenta) serotonin application. **(E)** Burst fraction (i.e., the fraction of action potentials belonging to bursts; see “Materials and Methods” section) was significantly lower before (left, black) as compared to after (right, magenta) serotonin application. *Indicates a statistically significant difference at the *p* = 0.05 level as indicated by a Wilcoxon’s signed-rank test.

We first investigated how serotonin application affected ELL pyramidal cell activity under baseline conditions (i.e., when the animal’s EOD was not modulated). To do so, extracellular recordings from ELL pyramidal cells were obtained (see “Materials and Methods” section) while a double-barrel pipette containing both glutamate and serotonin was advanced independently (Figure 2A). We used glutamate to ascertain that the double-barrel pipette was in the vicinity of the cell recorded from, as ascertained from short-latency increases in spiking activity when glutamate is ejected *via* air pressure (see “Materials and Methods” section). Serotonin was then applied *via* air pressure. Figure 2B (top, black trace, and large arrows) shows the recorded spiking activity from a typical ELL pyramidal cell before serotonin application. Spiking activity typically consisted of isolated spikes (Figure 2B, top, small arrows) and occasional bursts (Figure 2B, top, vertical bars). However, after serotonin application, this same cell displayed an increased tendency to fire bursts of action potentials (Figure 2B, bottom, magenta; compare with top panel). The spike train consisted mostly of bursts with larger length and much fewer isolated spikes (Figure 2B, bottom, vertical bars, and small arrows; compare with top panel). This change in excitability was also seen when plotting the distribution of ISIs (the time between consecutive action potentials; Figure 2C). We found that the ISI distribution was bimodal before (Figure 2C, black) and after (Figure 2C, magenta) and used the ISI corresponding to the trough between both modes as a threshold to separate the spike train into bursts and isolated spikes (see “Materials and Methods” section). Qualitatively similar results were seen across our dataset. Overall, serotonin application significantly increased both the mean firing rate (Figure 2D; control: 11.38 ± 5.78 Hz; serotonin: 16.36 ± 7.42 Hz; Wilcoxon’s signed-rank test, *P* < 0.001) and the burst fraction (i.e., the fraction of action potentials belonging to bursts; Figure 2E; control: 0.18 ± 0.15 Hz; serotonin: 0.37 ± 0.21 Hz; Wilcoxon’s signed-rank test, *P* < 0.01). There was no significant correlation between the change in burst fraction due to serotonin application and the cell’s firing rate before serotonin application (*r* = 0.05, *n* = 16, *p* = 0.87).

### Serotonin Application Increases ELL Pyramidal Cell Responses to Envelope Stimuli

**Figure 3:**
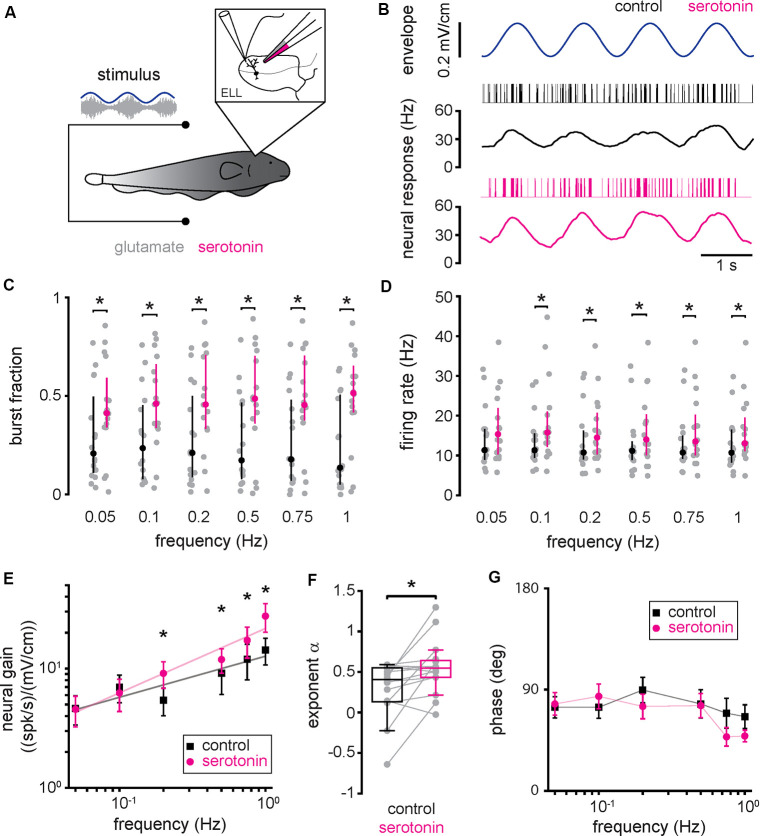
Serotonin application increases pyramidal neuron responses to envelope stimuli through increased burst firing. **(A)** Schematic showing a pyramidal cell that is being recorded from extracellularly while a double-barrel pipette containing both glutamate as well as serotonin is located in the vicinity. **(B)** Spike train (black vertical bars) and firing rate (black trace) responses from a typical pyramidal cell to an envelope stimulus (blue, top) before serotonin application. Also shown are the spike train (magenta vertical bars) and firing rate (magenta trace) responses from this same pyramidal cell to the envelope stimulus (blue, top) after serotonin application. **(C)** Burst fraction before (black) and after (magenta) serotonin application for all envelope frequencies tested. **(D)** Firing-rate before (black) and after (magenta) serotonin application for all envelope frequencies tested. **(E)** Population averaged sensitivity (i.e., neural gain) between the envelope stimulus and the full spike train before (black) and after (magenta) serotonin application as a function of temporal frequency. **(F)** Best-fit power-law exponents before (left, black) and after (right, magenta) serotonin application to the neural gain as a function of temporal frequency curves shown in panel **(E)**. **(G)** Population averaged phase between the envelope stimulus and the full spike train before (black) and after (magenta) serotonin application as a function of temporal frequency. Throughout, “*” indicates a statistically significant difference at the *p* = 0.05 level using either a Wilcoxon’s signed-rank test or a Student’s *t*-test.

Next, we investigated how serotonin application affected ELL pyramidal cell responses to envelope stimuli. To do so, we used the same setup to apply serotonin except that the same envelope stimuli were presented to each neuron before (i.e., control) and after serotonin application (Figure 3A). Envelope stimuli consisted of sinewaves with frequencies 0.05 Hz, 0.1 Hz, 0.2 Hz, 0.5 Hz, 0.75 Hz, and 1 Hz (see “Materials and Methods” section). The response of a typical ELL pyramidal cell before serotonin application to a sinusoidal envelope stimulus is shown in Figure 3B (top, black). ELL pyramidal cells typically increased their spiking activity near the local maxima of the envelope, such that there was a noticeable modulation in the time-dependent firing rate (Figure 3B, black). After serotonin application, there was increased burst firing near the local maxima of the envelope, such that the modulation in firing rate was greater (Figure 3B, magenta). Indeed, there were significant increases in both burst fraction (Figure 3C) and firing rate (Figure 3D) for all envelope frequencies tested. As the neural responses followed the stimulus waveform in an almost one-to-one fashion during both conditions, we used linear systems analysis to quantify the responses of ELL pyramidal cells to envelope stimuli. Specifically, we computed both the neural gain, which is the ratio of the amplitude of the firing rate modulation to that of the envelope stimulus and the phase, which is the amount of time by which one must shift the envelope stimulus to be in phase with the firing rate response relative to the stimulus period (see “Materials and Methods” section). Before the serotonin application, we found that neural gain increased as a power law as a function of increasing envelope frequency (Figure 3E, black), which is consistent with previous results (Martinez et al., 2016). After serotonin application, the neural gain was increased primarily for higher envelope frequencies (Figure 3E, magenta). This change was quantified by computing the best-fit power-law exponent to the neural tuning curves (i.e., the neural gain as a function of envelope frequency) before (Figure 3F, black) and after (Figure 3F, magenta) serotonin application. We found that serotonin application significantly increased the power-law exponent (Figure 3F, compare black and magenta; control: 0.30 ± 0.33; serotonin: 0.55 ± 0.32 Hz; Student’s *t*-test, *P* < 0.05). Overall, there was no significant correlation between the change in exponent due to serotonin application and the cell’s baseline firing rate before serotonin application (*r* = 0.27, *n* = 16, *p* = 0.32). When analyzing the phase relationship between the envelope stimulus and the firing rate response, we observed that it remained relatively constant and that it was not significantly altered by serotonin application (Figure 3G, compare black and magenta; Kruskal–Wallis test, chi-square = 0.94, *df* = 1, *P* = 0.01).

### Increased ELL Pyramidal Cell Responses to Envelope Stimuli Due to Serotonin Application Are Primarily Due to Increased Burst Firing

**Figure 4:**
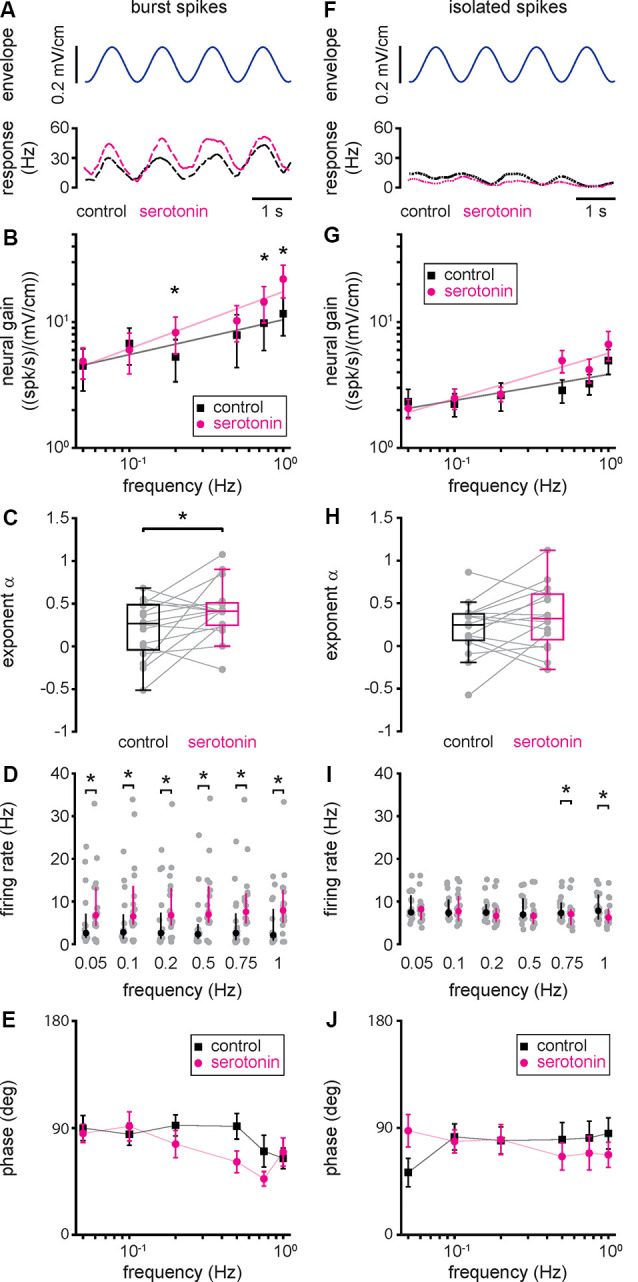
Changes in neural responses to envelope stimuli due to serotonin application are due to changes in burst firing. **(A)** Envelope stimulus (top, blue) and the firing rate responses (bottom) of the burst (long-dashed) spike train before (black) and after (magenta) serotonin application. **(B)** Population averaged sensitivity (i.e., neural gain) between the envelope stimulus and the burst spike train before (black) and after (magenta) serotonin application as a function of temporal frequency. **(C)** Best-fit power-law exponents before (left, black) and after (right, magenta) serotonin application. **(D)** Firing-rate before (black) and after (magenta) serotonin application for all envelope frequencies tested when using the burst train. **(E)** Population averaged phase between the envelope stimulus and the burst spike train before (black) and after (magenta) serotonin application as a function of temporal frequency. **(F)** Same as **(A)**, but for the isolated spike train (short-dashed). **(G)** Same as **(B)**, but for the isolated spike train. **(H)** Same as **(C)**, but for the isolated spike train. **(I)** Same as **(D)**, but for the isolated spike train. **(J)** Same as **(E)**, but for the isolated spike train. Throughout, “*” indicates a statistically significant difference at the *p* = 0.05 level using either a Wilcoxon’s signed-rank test or a Student’s *t*-test.

To gain a better understanding as to how serotonin application led to increased ELL pyramidal cell responses to envelope stimuli, we separated the spike train into the burst and isolated spike trains (see “Materials and Methods” section). Our results show that, when considering only the burst train, there was increased rate modulation after serotonin application (Figure 4A), as observed for the full spike train. As such, neural gain computed from the burst train was increased after serotonin application primarily for higher envelope frequencies (Figure 4B, compare black and magenta). Thus, serotonin application led to a significant increase in the best-fit power-law exponent (Figure 4C, compare black and magenta, control: 0.20 ± 0.33 Hz; serotonin: 0.42 ± 0.32 Hz; Student’s *t*-test, *P* < 0.05). Overall, serotonin application significantly increased the firing rate computed from the burst train (Figure 4D, compare black and magenta). We found no significant change in the phase (Figure 4E, compare black and magenta, Kruskal–Wallis test, chi-square = 1.45, *df* = 1, *P* = 0.23).

Qualitatively different results were observed when instead considering the isolated spike train (Figure 4F). Indeed, the rate computed from the isolated spike train displayed much weaker modulation before serotonin application than those obtained from the burst train or the full spike train (compare short-dashed black trace in Figure 4F to long-dashed black trace in Figure 4A and solid black trace in Figure 3B, respectively). Moreover, after serotonin application, there was no increase in modulation when considering the isolated spike train (Figure 4F, compare black and magenta short-dashed traces). As such, serotonin application did not lead to significant increases in either of neural gain (Figure 4G, compare black and magenta), best-fit power-law exponent (Figure 4H, compare black and magenta, control: 0.21 ± 0.31 Hz; serotonin: 0.35 ± 0.36 Hz; Student’s *t*-test, *P* = 0.12), firing rate computed from the isolated spike train (Figure 4I, compare black and magenta), or phase (Figure 4J, compare black and magenta, Kruskal–Wallis test, chi-square = 0.78, *df* = 1, *P* = 0.38). We thus conclude that the changes in the neural gain observed after serotonin application are primarily, if not exclusively, due to changes in the burst rather than isolated spike firing.

### Serotonin Application Increases the Jamming Avoidance Response

**Figure 5:**
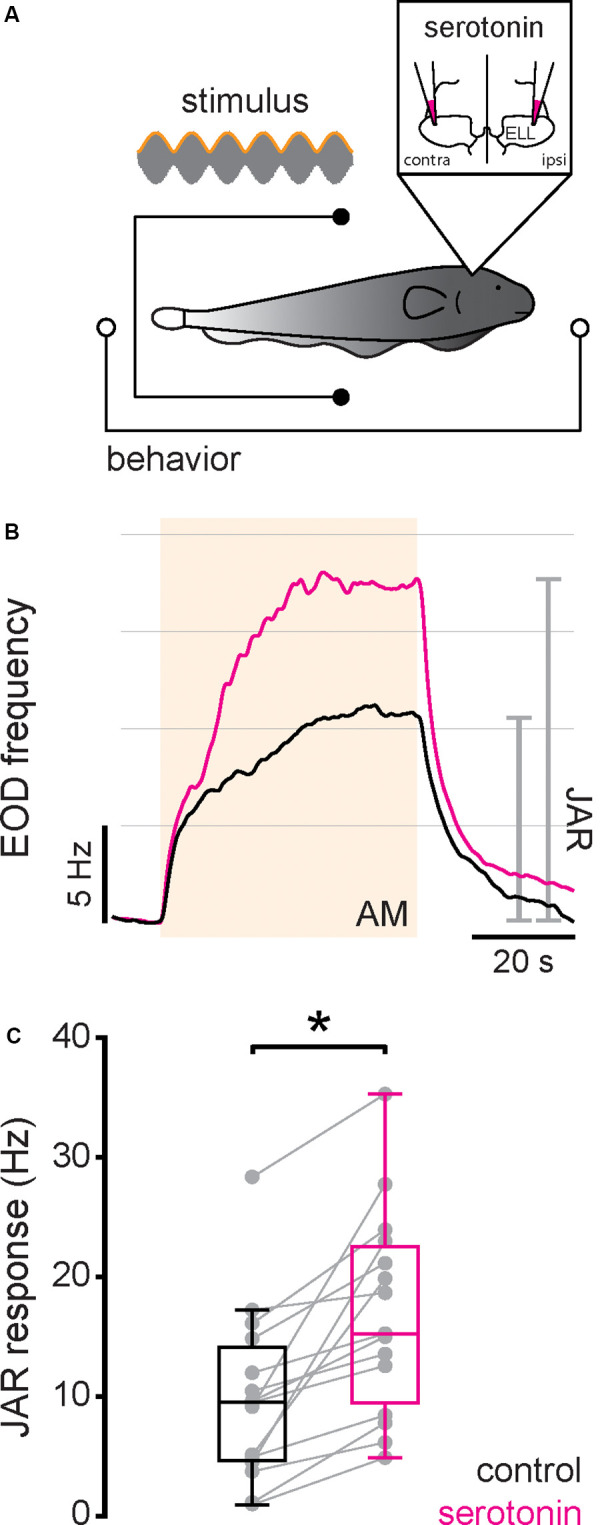
Serotonin application increases the jamming avoidance response (JAR) response. **(A)** Schematic showing the setup in which the animal’s behavior (EOD) was recorded while being stimulated and serotonin injected bilaterally in the ELL. **(B)** Time course of the EOD frequency for a typical fish before (black) and after (magenta) serotonin application. The stimulus was a 4 Hz sinusoidal AM (see “Materials and Methods” section). The shaded band indicates the period during which the stimulus was applied. The JAR magnitude is measured as the change in EOD frequency from immediately before the stimulus onset to immediately before stimulus offset (gray lines). **(C)** Jar magnitude before (black) and after (magenta) serotonin application. Serotonin application significantly increased the JAR magnitude (Student’s *t*-test, *P* < 0.001) as indicated by the “*”.

Next, we investigated the effects of serotonin application on behavioral responses. To do so, serotonin was applied bilaterally into the ELL while the animal was being stimulated and behavioral responses recorded (Figure 5A; see “Materials and Methods” section). We first studied the effects of serotonin application in the extensively studied JAR. The JAR consists of an increase in EOD frequency during stimulation caused by interference of signals with similar frequencies (Hitschfeld et al., 2009; Deemyad et al., 2013) and it has been shown previously that serotonin increases the JAR magnitude in *A. leptorhynchus*. Thus, we compared the magnitude of the JAR before and after serotonin application using a 4 Hz sinusoidal AM stimulus (see “Materials and Methods” section). We found that serotonin application was indeed effective, as the JAR magnitude was increased after serotonin application in both an example fish (Figure 5B, compare black and magenta) and significantly across our dataset (Figure 5C, compare black and magenta, control: 9.87 ± 6.99 Hz; serotonin: 16.90 ± 8.26 Hz; Student’s *t*-test, *P* < 0.001).

### Serotonin Application Increases Behavioral Responses to Envelope Stimuli

**Figure 6:**
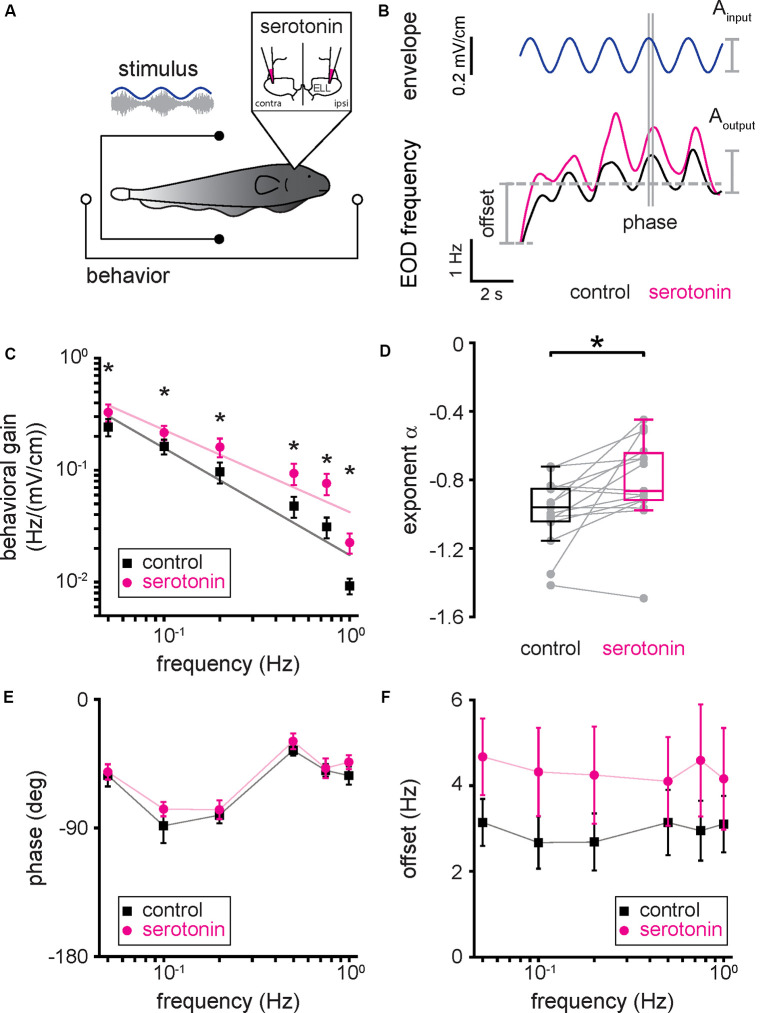
Serotonin application increases behavioral responses to envelope stimuli. **(A)** Schematic showing the setup in which the animal’s behavior (EOD frequency) was recorded while being stimulated and serotonin injected bilaterally in the ELL. **(B)** EOD frequency from a typical fish before (bottom, black) and after (bottom, magenta) serotonin application to the same envelope stimulus (top, blue). The behavioral response was quantified using the behavioral gain, which is the ratio of the EOD frequency modulation A_output_ to that of the stimulus A_input_. The behavioral response was also quantified using the phase, which is the time by which the behavioral response is shifted concerning the envelope stimulus normalized by the stimulus cycle, and the offset, which is the increase in the mean EOD frequency during stimulation. **(C)** Population averaged behavioral gain between the envelope stimulus and the EOD frequency before (black) and after (magenta) serotonin application as a function of envelope frequency. **(D)** Best-fit power-law exponents before (left, black) and after (right, magenta) serotonin application to the behavioral gain as a function of temporal frequency curves shown in panel **(C)**. **(E)** Population averaged phase between the envelope stimulus and the EOD frequency before (black) and after (magenta) serotonin application as a function of temporal frequency. **(F)** Population averaged offset between the envelope stimulus and the EOD frequency before (black) and after (magenta) serotonin application as a function of temporal frequency. Throughout, “*” indicates a statistically significant difference at the *p* = 0.05 level using either a Wilcoxon’s signed-rank test or a Student’s *t*-test.

We next tested whether and, if so, how serotonin application affects behavioral responses to envelope stimuli. To do so, we presented the same envelope stimuli used for electrophysiology before and after applying serotonin bilaterally into the ELL (Figure 6A). Figure 6B shows a representative EOD frequency change (bottom) in response to the envelope stimulus (top, blue) before (black) and after (magenta) serotonin application. Before the serotonin application, the behavioral response consisted of an overall increase in the EOD frequency which was modulated quasi-sinusoidally (Figure 6B, black), consistent with previous results (Metzen and Chacron, 2014; Martinez et al., 2016). After serotonin application, we found that the EOD frequency was greater overall during stimulation and displayed greater quasi-sinusoidal modulations that follow the stimulus waveform (Figure 6B, magenta). As such, we used linear systems identification techniques to quantify the behavioral response. Specifically, we computed the behavioral gain as the ratio of the EOD frequency modulation amplitude (i.e., “A_output_”) to that of the stimulus (i.e., “A_input_”). We also computed the phase, which is the amount of time by which the envelope stimulus must be shifted to be in phase with the EOD frequency, as well as the offset, which was computed as the difference between the mean EOD frequency during the stimulus and that before stimulus onset (see Figure 6B and “Materials and Methods” section). Before the serotonin application, the behavioral gain decreased as a power law with increasing envelope frequency (Figure 6C, black), consistent with previous results (Martinez et al., 2016). However, serotonin application led to a greater increase in behavioral gain for higher envelope frequencies (Figure 6C, compare black and magenta). As such, the best-fit power-law exponent increased after serotonin application (Figure 6D, compare black and magenta; control: −0.99 ± 0.19 Hz; serotonin: −0.80 ± 0.25 Hz; Student’s *t*-test, *P* < 0.05), such that the behavioral gain decreased less steeply with increasing envelope frequency (Figure 6C, compare black and magenta). We observed no significant change in phase (Figure 6E, compare black and magenta; Kruskal–Wallis test, chi-square = 0.41, *df* = 1, *P* = 0.52) and an overall increased offset (Figure 6F, compare black and magenta; Kruskal–Wallis test, chi-square = 8.31, *df* = 1, *P* < 0.05) after serotonin application.

### Changes in Burst Firing Due to Serotonin Application Best Correlate With the Resulting Changes in Behavior

**Figure 7:**
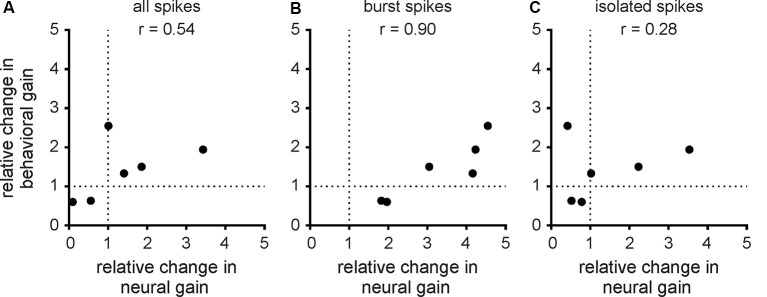
Changes in behavioral gain best correlate with changes in the neural gain of bursts. **(A)** Population-averaged relative change in behavioral gain as a function of the population-averaged relative change in neural gain from the all spike train. The data points correspond to the different envelope frequencies. There was no significant correlation between both quantities (*r* = 0.54, *n* = 6, *p* = 0.27). **(B)** Same as **(A)**, but for the burst spike train. There was a significant correlation between both quantities (*r* = 0.90, *n* = 6, *p* < 0.05). **(C)** Same as **(A)**, but for the isolated spike train. There was no significant correlation between both quantities (*r* = 0.28, *n* = 6, *p* = 0.59).

Finally, we investigated the relationship between changes in neural and behavioral responses due to serotonin application. To do so, we computed the relative change in behavioral gain for the different envelope frequencies and correlated this quantity with the relative change in neural gain when considering all spikes (Figure 7A; *r* = 0.54, *n* = 6, *p* = 0.27), burst spikes (Figure 7B; *r* = 0.90, *n* = 6, *p* < 0.05), and isolated spikes (Figure 7C; *r* = 0.28, *n* = 6, *p* = 0.59). Overall, we found a significant correlation only for burst spikes, which suggests that bursts rather than isolated spikes are being decoded downstream to help generate behavioral responses. This is further discussed below.

## Discussion

### Summary of Results

We investigated the effects of serotonin on both lateral segment ELL pyramidal cell and behavioral responses to envelope stimuli in the weakly electric fish *A. albifrons*. Serotonin application increased ELL pyramidal cell excitability and promoted burst firing under baseline conditions. In response to envelope stimuli, serotonin application increased the response gain, such that the envelope stimulus gave rise to greater modulation in firing rate. There was a greater increase in gain for high envelope frequencies, such that neural gain increased more steeply as a function of envelope frequency, as quantified by a greater power-law exponent. Such changes in responsiveness were primarily, if not exclusively, due to increased burst firing. This is because serotonin application did not lead to significant changes in responsiveness when considering the isolated spike train. Finally, we investigated the effects of serotonin application on behavioral responses. Serotonin application increased behavioral gain, such that envelope stimuli gave rise to greater modulations in EOD frequency. There was a greater increase in behavioral gain for greater envelope frequencies, such that behavioral gain decreased less steeply as a function of increasing frequency after serotonin application, as quantified by a power-law exponent with decreased magnitude. Overall, changes in behavioral gain as a function of frequency were best correlated with changes in neural gain when considering bursts, rather than the full spike train or isolated spikes.

### Effects of Serotonin on Sensory Processing: Comparison Between *A. albifrons* and *A. leptorhynchus*

Our results have shown that serotonin application increased lateral segment ELL pyramidal cell burst activity in *A. albifrons*, such that the neural gain increased more steeply with increasing frequency as characterized by a greater exponent. Previous results have shown that, before serotonin application, the tuning curves of lateral segment ELL pyramidal cells to envelope stimuli were essentially identical in both *A. albifrons* and *A. leptorhynchus* (Huang and Chacron, 2016; Huang et al., 2016; Martinez et al., 2016). The effects of serotonin application on ELL pyramidal cell responses to envelope stimuli have been recently characterized by us in *A. leptorhynchus* (Marquez and Chacron, 2020b). In *A. leptorhynchus*, serotonin application also increases ELL pyramidal cell excitability and responses to envelope stimuli, primarily through increased burst firing. The changes in neural gain observed for *A. albifrons* in the current study were similar to those previously observed for *A. leptorhynchus* (compare Figures 3E, 4B,G with Figures 4D–F of Marquez and Chacron, 2020b, respectively). As such, we hypothesize that serotonin increases ELL pyramidal cell excitability and responsiveness through the same mechanisms in both *A. albifrons* and *A. leptorhynchus*. Specifically, serotonin increases ELL pyramidal neuron excitability in *A. leptorhynchus* by inhibiting potassium channels, namely small conductance calcium-activated and M-type channels, that both give rise to an afterhyperpolarization after the action potential (Deemyad et al., 2011, 2013; for review see Márquez et al., 2013; Huang and Chacron, 2017). The removal of this afterhyperpolarization allows for the depolarizing afterpotential, which is critical for burst generation in ELL pyramidal cells, to potentiate during the burst, thereby increasing the number of spikes during the burst (Lemon and Turner, 2000; Krahe et al., 2008; Toporikova and Chacron, 2009; for review see Metzen et al., 2016). Further studies carried out in *A. albifrons* are however needed to verify these predictions.

Our results have shown that serotonin application increases the power-law exponent characterizing the relationship between neural gain and envelope frequency (i.e., the “neural exponent”) in *A. albifrons*. Previous studies carried out in *A. leptorhynchus* have shown that the neural exponent is closely matched to the statistics of natural envelope stimuli such as to optimally encode them *via* temporal whitening (Huang and Chacron, 2016; Huang et al., 2016; for review see Huang and Chacron, 2017; Metzen and Chacron, 2019). Specifically, this is because the power-law increase in neural sensitivity compensates for the decaying spectral power of natural envelope stimuli with increasing envelope frequency, such that the resulting response power is independent of frequency (i.e., “temporally whitened”). Further studies have shown that descending pathways play a critical role in shaping the envelope tuning curve of ELL pyramidal cells (Huang et al., 2018). Most recently, it was shown that serotonin was critical in mediating adaptive optimization of envelope stimuli in *A. leptorhynchus* (Huang et al., 2019). Specifically, when the statistics of the envelope stimulus change, processes occur that cause changes in the response properties of ELL pyramidal cells, such that their responses to the new envelope stimulus become more temporally whitened over time. Huang et al. (2019) showed that serotonin is necessary to achieve such sensory adaptation. Moreover, sensory adaptation is seen ubiquitously across systems and species (Wark et al., 2007; Sharpee et al., 2014). Thus, we hypothesize that similar mechanisms are found in *A. albifrons*. However, further studies are needed to ascertain whether natural envelope stimulus statistics (i.e., how does spectral power decay with increasing envelope frequency) are similar in both *A. albifrons* and *A. leptorhynchus* to validate this hypothesis.

### Effects of Serotonin on Behavioral Responses: Comparison Between *A. albifrons* and *A. leptorhynchus*

Previous studies have shown that both *A. leptorhynchus* and *A. albifrons* display nearly identical responses to envelopes (Metzen and Chacron, 2014, 2015; Martinez et al., 2016; Thomas et al., 2018). Specifically, in both species, the EOD frequency tracks the detailed timecourse of the envelope stimulus. Such behavioral responses habituate to repeated stimulus presentations and are thus thought to be mediated by higher brain centers. In both species, behavioral gain decays as a power-law with increasing envelope frequency. In *A. leptorhynchus*, the power-law exponent is the same as that describing how the stimulus spectral power decays with increasing envelope frequency (Metzen and Chacron, 2014). This match between behavioral gain and stimulus spectral power is thought to optimize behavioral responses to maximize responses to the frequency components that are most prevalent in the stimulus. This is supported by results showing that, in response to envelope stimuli with different statistics (i.e., different power-law exponents), behavioral responses in *A. leptorhynchus* adapt such that the behavioral exponent better matches that of the stimulus (Huang et al., 2019). The fact that both *A. leptorhynchus* and *A. albifrons* display nearly identical behavioral responses to envelope stimuli before serotonin application suggests that these responses serve a similar function and that they are mediated by similar mechanisms in the brain.

However, a comparison of our results and previous ones obtained for *A. leptorhynchus* reveals an important difference. Indeed, while our results have shown that serotonin application increases behavioral gain more for high envelope frequencies than for low envelope frequencies (Figure 6C), previous results have shown that, for *A. leptorhynchus*, behavioral gain increases more for low envelope frequencies than for high envelope frequencies (see Figure 7E in Marquez and Chacron, 2020b). Thus, after serotonin application, the behavioral gain decreases less steeply with increasing envelope frequency in *A. albifrons* but more steeply for *A. leptorhynchus*. Indeed, while the behavioral exponent increases for *A. albifrons* (Figure 6D), this same exponent decreases for *A. leptorhynchus* (see inset of Figure 7E in Marquez and Chacron, 2020b). Because serotonin application gave rise to similar effects on ELL pyramidal cell activity in both species, these qualitative differences on behavior cannot be ascribed to differences in the spiking activities of ELL pyramidal cells. Rather, we hypothesize that the observed differences in behavior result from differences in decoding of ELL pyramidal cell activity by higher brain areas in both species. In particular, the behavioral responses to envelope stimuli are likely in part mediated by the forebrain, which has recently been the focus of intense investigation in *A. leptorhynchus* (Giassi et al., 2012a,b,c; Trinh et al., 2016; Wallach et al., 2018; Fotowat et al., 2019). Further comparative studies carried out in *A. albifrons* are needed to ascertain as to whether and, if so, how the envelope responses of ELL pyramidal cells are decoded differentially by higher brain centers.

Future studies should also focus on how midbrain neurons, which receive input from ELL neurons, respond to envelope stimuli. Such responses have been investigated in part in *A. leptorhynchus* (McGillivray et al., 2012) but not at all in *A. albifrons*. However, based on the relative similarity of midbrain neural responses observed in *A. leptorhynchus* (Chacron et al., 2009; Chacron and Fortune, 2010) and in *Eigenmannia virescens* (Fortune and Rose, 1997a,b, 2001, 2003; Rose and Fortune, 1999), we would expect that midbrain neurons would respond similarly to the envelope in both *A. leptorhynchus and A. Albifrons*. In particular, we hypothesize that the subthreshold membrane conductances displayed by midbrain neurons (Fortune and Rose, 1997a; Rose and Fortune, 1999; Chacron and Fortune, 2010) enable them to better detect coincident burst firing from ELL pyramidal cell populations after serotonin application. Indeed, burst firing from ELL pyramidal cells would serve to generate a stronger signal that would better counteract the depression observed at ELL-midbrain synapses (Rose and Fortune, 1999; Chacron et al., 2009). Further studies are however needed to test this prediction.

What are the functional implications of serotonin primarily increasing behavioral responses to envelopes for low temporal frequencies in *A. leptorhynchus* and primarily for higher temporal frequencies in *A. albifrons*? As mentioned above, these give rise to behavioral sensitivities that are characterized by different exponents. We hypothesize that these might be better matched to different envelope statistics in both species. Indeed, a recent study has shown that the level of activity in *A. leptorhynchus* strongly influences the exponent at which the envelope stimulus power decays (Huang et al., 2019). As such, it is conceivable that different movement strategies in *A. leptorhynchus* and *A. albifrons* would give rise to different statistics (i.e., envelope stimulus power decays with different exponents), and that serotonin would attempt to better match behavioral sensitivity with these differing statistics, thereby explaining the different effects on behavior. Further studies are however needed to test this hypothesis.

Our results have shown that serotonin application increased JAR magnitude in *A. albifrons*. Overall, our results were similar to those obtained previously for *A. leptorhynchus* (Deemyad et al., 2013; Marquez and Chacron, 2020b). At first glance, this would suggest that the effects of serotonin on the JAR would serve a similar function in both species. It has been observed that EOD frequency is a signal of social dominance in *A. leptorhynchus* and other apteronotid species, with males having EOD frequencies higher than females and more dominant males having higher EOD frequencies than less dominant ones (Triefenbach and Zakon, 2008; Fugère and Krahe, 2010; Fugère et al., 2011; Henninger et al., 2018; Raab et al., 2019). Indeed, injection of androgens such as 11-ketotestosterone will increase EOD frequency in *A. leptorhynchus* (Meyer et al., 1987; Schaefer and Zakon, 1996). Results showing that serotonin application increased JAR magnitude in *A. leptorhynchus* are thus consistent with the JAR as a means of establishing social hierarchy in a group of fish (for review see Rose, 2004). More submissive male individuals tend to display greater levels of serotonin following aggressive encounters (Larson and Summers, 2001), and these greater levels would be expected to give rise to a larger JAR when encountering another conspecific, thereby making that submissive individual appear stronger.

However, the situation appears to be qualitatively different when considering *A. albifrons*. This is because, contrary to *A. leptorhynchus*, male *A. albifrons* tend to have lower EOD frequencies than females (Zakon and Dunlap, 1999) and because androgen treatment actually decreases EOD frequency (Dunlap et al., 1998). These results suggest that, in male *A. albifrons*, a lower EOD frequency is a signal of increased dominance. As such, our results showing that serotonin application increased the magnitude of the JAR in *A. albifrons* are a bit counterintuitive in light of the interpretation made above for *A. leptorhynchus*. This is because increased serotonin levels in a submissive male would then make this individual appear even more submissive when encountering another conspecific. Thus, it is conceivable that the function of serotonin towards determining JAR behavior is qualitatively different in *A. albifrons* and *A. leptorhynchus*. Alternatively, it is important to recall that both these species are not very gregarious, as they tend to be found by themselves most of the time (Stamper et al., 2010). As such, the JAR might not play an important role in establishing social dominance. Further studies are needed to better understand the functional role of the JAR in both species.

### Implications for Other Systems

Here we used a comparative approach to gain a better understanding as to the function of the serotonergic system on sensory processing. Such approaches have proven useful to distinguish brain coding strategies that can be generalized from those that are species-specific (Carlson, 2012; Hale, 2014; Brenowitz and Zakon, 2015). In the case of the serotonergic system, the remarkable conservation across vertebrate species suggests a common function (Parent, 1981). While this may be true in general, our results show that even when comparing two very closely related species such as *A. leptorhynchus* and *A. albifrons*, application of serotonin in the same brain area can give rise to opposite effects on behavioral responses. It is thus likely that the serotonergic system will serve species-specific functions. Further studies are needed to ascertain whether this hypothesis holds across different sensory systems and species.

## Data Availability Statement

All datasets presented in this study are included in the article.

## Ethics Statement

The animal study was reviewed and approved by McGill University Downtown facility B animal care committee.

## Author Contributions

MM and MC: conceptualization, methodology, validation, writing—review, and editing. MM: data curation, formal analysis, investigation, and visualization. MC: funding acquisition, project administration, resources, software, supervision and writing—original draft.

## Conflict of Interest

The authors declare that the research was conducted in the absence of any commercial or financial relationships that could be construed as a potential conflict of interest.
